# A unifying theory of aging and mortality

**DOI:** 10.1038/s41598-025-11454-4

**Published:** 2025-08-06

**Authors:** Valentin Flietner, Bernd Heidergott, Frank den Hollander, Ines Lindner, Azadeh Parvaneh, Holger Strulik

**Affiliations:** 1 PwC, Bernhard-Wicki-Strasse 8, 80636 Munich, Germany; 2 Department of Operations Analytics, VU University Amsterdam, De Boelelaan 1105, 1081 HV Amsterdam, The Netherlands; 3 Leiden University, Mathematical Institute, Einsteinweg 55, 2333 CC Leiden, The Netherlands; 4 Department of Economics, VU University Amsterdam, De Boelelaan 1105, 1081 HV Amsterdam, The Netherlands; 5 Department of Economics, University of Göttingen, Platz der Göttinger Sieben 3, 37073 Göttingen, Germany

**Keywords:** Biological models, Applied mathematics

## Abstract

In this paper, we advance the network theory of aging and mortality by developing a causal mathematical model for the mortality rate. First, we show that in large networks, where health deficits accumulate at nodes representing health indicators, the modelling of network evolution with Poisson processes is universal and can be derived from fundamental principles. Second, with the help of two simplifying approximations, which we refer to as mean-field assumption and homogeneity assumption, we provide an analytical derivation of Gompertz law under generic and biologically relevant conditions. Third, we identify for which network parameters Gompertz law is accurate, express the parameters in Gompertz law as a function of the network parameters, and illustrate our computations with simulations and analytic approximations. Our paper is the first to offer a full mathematical explanation of Gompertz law and its limitations based on network theory.

## Background and challenges

### Motivation

Why do we die when we get old? Traditionally, it is understood that we eventually die of ‘old age’. In scientific terms, the mortality rate *m*(*t*) at age *t* describes the probability that a person dies in a short age interval following age *t*, namely, for $0 < \Delta \ll 1$ the probability of death during the time interval $[t,t+\Delta )$ given that no death occurred during the time interval [0, *t*] equals $m(t)\Delta$. The seminal paper by Mitnitski, Rutenberg, Farrell and Rockwood^16^ led to a paradigm shift in the interpretation of the age-dependency of the mortality rate (see also^4,22,28^). The mortality rate is modeled by a *dynamic network*, in which nodes represent health indicators, the dynamic relationship between the states of the nodes models the interdependency of the health indicators, and death is defined as the time it takes to reach a network state in which two carefully selected nodes, called *mortality nodes*, are both damaged. The model showed a good fit to actual mortality rate data and offered a new interpretation of death as the accumulation of damaged health indicators. A drawback, however, is that a network approach to aging and mortality typically requires the identification of an appropriate network structure through numerical experiments in a trial-and-error process.

The universality of Gompertz law calls for an explanatory model. In a recent paper^18^, a proposal is made to explain Gompertz law with the help of a simple mean-field argument. Specifically, death is modelled by a small fraction of nodes being in the damaged state (where the actual fraction used in the model is a design parameter). The aggregate model in^18^ does not provide an explicit modelling of death, nor of the network structure as is done in the Mitnitski et al. model, where death is modelled via the mortality nodes, which are the two most highly connected nodes of the network. Moreover, the model in^18^ requires that a very small fraction of nodes is initially in the failed state. The key argument in^18^ is that a simple ordinary differential equation models the evolution of the mean number of failed nodes over time, the solution of which yields the structure of Gompertz law.

More generally, our paper contributes to the an extended literature that aims for a theoretical foundation of Gompertz law. An early attempt is provided by^25^, based on the assumption of finite biological capacity or reserve that declines with age. A key implication is the so-called Strehler-Mildvan correlation, a negative and log-linear association between the Gompertz parameters ($\alpha$ and $\beta$ in our notation). We improve on^25^ by offering a theory based on much less restrictive assumptions. In particular, the basic assumption in^25^ that human vitality is challenged by exponentially distributed stress events has been criticised as unjustified and as an ad-hoc implementation of desired exponentiality into the model^7^. In contrast, our approach offers a structurally causal model by deriving mortality from principles based on the interaction of health deficits within a networked system. Furthermore, our theory does not depend on the validity of the Strehler-Mildvan correlation. The Strehler-Mildvan correlation implies the rectangularisation of survival curves and the constancy of human life span^9^, an implication that is hard to square with modern longevity research suggesting that human life span can be extended by medical progress. The universality of the Strehler-Mildvan correlation has also been challenged in empirical studies, diagnosing its breakdown in recent data and noting increasing human life span^27,29–31^. The universality of Gompertz law, in contrast, remains firmly established.

An earlier mathematical theory of aging and mortality was developed by adapting reliability theory to living systems^9,10^. Like network-based models, it conceptualises the organism as a system composed of multiple components vulnerable to random damage over time. Both frameworks assume that the organism can tolerate some degree of damage, but survival is compromised once damage exceeds a critical threshold. However, reliability theory treats the components as independent units with fixed failure rates, and system failure (death) occurs when a predefined number of components fail. It does not account for interactions among components. In contrast, network theories of aging model the organism as a system of interdependent nodes, where the state of each node is influenced by the states of its neighbours. This allows damage to propagate through the network – if surrounding nodes are compromised, then a node becomes more likely to fail. This feature captures better how local damage spreads and leads to widespread decline. The modelling of dynamic and interacting damage and repair processes – instead of fixed hazard rates – is better aligned with the biological understanding of aging and resilience^1,21^. Furthermore, network models incorporate dynamic interactions between damage and repair, and offer a more biologically realistic representation of aging and resilience than static hazard rates^1,21^.

In the present study, we extend the network approach by moving from simulation-based modelling to a robust and foundational framework, and derive a causal mathematical model for the mortality rate under generic conditions. Our holy grail is to provide a mathematical derivation of *Gompertz law* for *m*(*t*), which is believed to be valid when *t* is neither too small nor too large. Before we state the mathematical model and identify our research question, we discuss in more depth the interpretation of the concept of ‘node’ and ‘state of a node’. Using results from network science, we explain why a certain network structure and a certain network dynamics can be argued for in the model.

The results of our study advance the theoretical understanding of the age-dependency of the mortality rate. Our analytical approach relies solely on very general structural assumptions about the network and supports the network choice made in^16^. This means that results that were previously derived from an ad-hoc fitting of a particular network structure are here derived as closed-form analytical solutions under broad and biologically relevant conditions.

Recent studies have shown that in humans (and other animals) damage at the molecular level, such as DNA methylation, protein glycoxidation and carbamylation, accumulates approximately linearly with age^5,12,14^. Reconciling this observation with the fact that in Gompertz law the rate of mortality rises exponentially with age has been considered an unsolved problem, and a scientific challenge in the biology of aging. In the present paper, we show how this conundrum can be resolved when molecular damage accumulation is associated with the average fraction of damaged nodes in a health network. Specifically, we show how Gompertz law is consistent with linear damage accumulation at the molecular level over a relevant age range.

### Gompertz law

**Figure 1:**
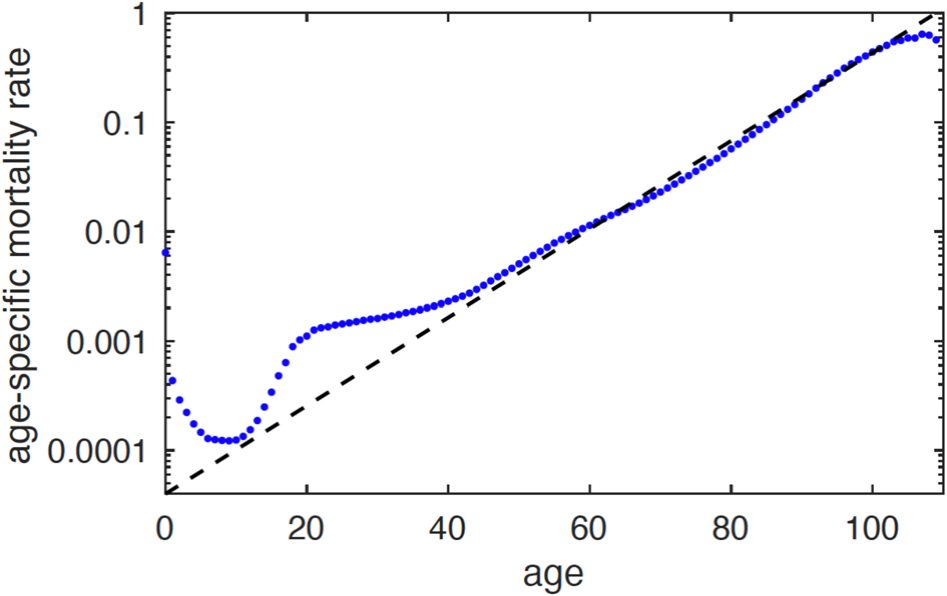
Age-specific mortality rate for US men in the period 2010–2019. Dots indicate data points taken from^15,17^. The straight line shows the Gompertz estimate $m({\textrm{age}}) = \alpha \,\textrm{e}^{\beta \times {\textrm{age}}}$ with $\alpha \approx 5.8 \times 10^{-5}$ and $\beta \approx 8.5 \times 10^{-2}$, and with age measured in years. The plot is semi-logarithmic (The data beyond 100 years may be less reliable because of measurement errors and selection effects.).

Human life can be roughly divided into two periods: a *phase of initial development* (ranging from birth to puberty), during which the mortality rate decreases, followed by a *phase of aging* (after puberty), during which the mortality rate increases. Figure 1 illustrates these two phases for US men in the period 2010–2019. It indicates that the phase of aging runs from 10 to 100 years of age, and that from 40 years of age onwards the mortality rate and age are log-linearly related. This empirical relationship, first observed and stated in 1825 by actuary Benjamin Gompertz^11^, is given by ($\approx$ means approximately)

1 $$\begin{aligned} m(t) \approx \alpha \, \textrm{e}^{\beta t}, \qquad t \ge 0, \end{aligned}$$

with parameters $\alpha ,\beta >0$, and is since referred to as *Gompertz law*.

Figure 1 illustrates Gompertz law by showing it together with the mortality rate of US men in the period 2010-2019. The figure indicates two *overshooting phases* relative to the Gompertz law, one between birth and 10 years of age and one between 10 and 40 years of age. Newborns are more likely to die of diseases that are far from fatal for grown-ups, such as gastroenteritis. An explanation is offered by health deficit models due to a lack of redundancy, such as organ reserves^3,24^. The lowest mortality is reached in puberty, when the body is fully developed and aging starts to take over from growth. From a mere physiological point of view, there is no explanation for the second overshooting phase, after puberty. Rather, this peak likely stems from other influences, such as risk preference, suicide, or war^26^. Note that (1) shows no barrier for increasing age, and hence refutes the frequently made assumption of a bounded life time. The fact that the sample size of all people who ever lived on earth increases continuously simply implies that the maximum ever-observed life length will keep on rising as time proceeds^6^.

The Gompertz parameters differ across sexes and across countries. On average across countries, women face a lower $\alpha$ and a higher $\beta$^9^, i.e., women have an initial advantage of a lower rate of aging that men eventually catch up with when getting older. Economically advanced countries are characterised by a lower $\alpha$ and a higher $\beta$, i.e., a lower initial mortality rate and a faster speed of aging^27^. A lower $\alpha$ can be associated with better (initial) physiological conditions, such as nutrition. The time invariance of the Gompertz law suggests that, correcting for country-specific and sex-specific background risk, humans share a common mechanism of aging, a common stochastic process according to which individual bodies lose function over time and bodily failures and health deficits accumulate.

### Features of health in an aging network

Medicine as a discipline is almost as old as mankind itself. Even though over the centuries medicine has undergone major paradigm shifts, a body of knowledge has emerged on how to assess the health status of a person, based on a few key health indicators such as blood pressure, sugar and cholesterol levels, heart rate, physical mobility, etc. With the advent of modern technology, more and more health indicators have become available. While such health indicators increase the knowledge on the health state, their added value is limited when measurements become more and more specific. Physicians need a small number of measurements to act on and, through centuries of medical practice, a few *central measurements* have emerged. The fact that these measurements represent the health state of the body fairly well indicates their predictive power.

Modelling the health indicators as *nodes* in a graph and their interconnectedness as *links* in the graph motivates us to assume that the network is *scale-free*, i.e., its empirical degree distribution approximately follows a power law. Furthermore, the *hubs* of the networks should be separated from each other, so that they can serve as a proxy of the network state in the local clusters that they dominate. This makes it reasonable to suppose that the network is *disassortative*, i.e., its degrees tend to be negatively correlated. We distinguish between *mortality nodes* and *aging nodes*. The former (small in number) are the health indicators that play a dominant role in the cause of death, while the latter (large in number) are the health indicators that form the overall network structure. Each node can be in a *healthy state* or a *damaged state*.

The model allows for a more speculative interpretation by taking the graph as a representation of the interconnectedness of physical parts of the body. In this interpretation, the mortality nodes represent the most vital organs, and the graph structure reflects the fact that certain physiological aspects of the body are more related to certain vital organs than others. Dissortativeness comes into play as a way of separating the different vital hubs as much as possible, so that if one hub is in a state of bad health, then its illness is contained as much as possible. The similarity with security structures of computer networks, or protocols for containing spread of diseases in infection models, is obvious.

The assumptions (*A*) and questions (*Q*) outlined below provide a framework for guidance.

- *A:* The aging nodes and the mortality nodes serve as predictors of the overall state of the network. *Q:* Can we show that when the mortality nodes reach the damaged state, a certain fraction of the aging nodes is damaged as well? Can we estimate this fraction?
- *A:* Scale-freeness and disassortativity are essential and rely on a hierarchy of health indicators. *Q:* To what extent is the presence of hubs crucial for predictability? If we decrease the out-degree of the hubs, then does the predictability of these hubs decrease?

The goal of the present paper is to propose a *causal* model that allows for a *data science approach* to mortality. In Sect. "Basic model" we define the basic model. In Sect. "Mathematical analysis" we present our analysis of this model. In Sect. "Simulations and numerical results" we illustrate the results with simulations and in Sect. "An analytic approximation" we provide an analytical approximation of Gompertz law. Section "Conclusion" concludes.

## Basic model

### Aging-network process

**Figure 2:**
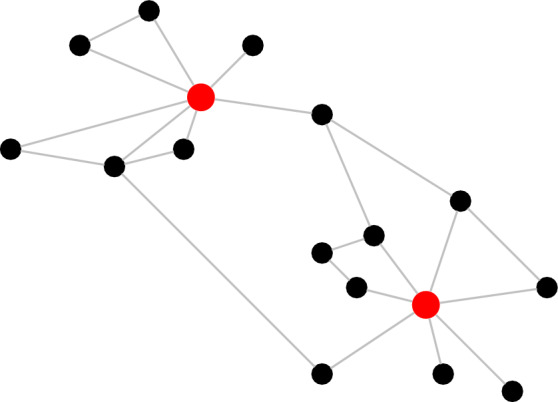
An example of a health network. The aging nodes are black, the mortality nodes are red.

We consider a graph *G* consisting of *n* nodes and certain links between these nodes, such that for each node *i* the set *N*(*i*) of neighbours of *i* is non-empty. Each node has a state that is either 0 (= healthy) or 1 (= damaged). A node represents a data measurement of the body. It is conceivable that there are limitless measurement possibilities for the body and that these have some causal interference structure. Since this structure is not known, we replace it by a graph. In Fig. 2 this graph is depicted, where the black nodes represent the aging nodes and the red nodes the mortality nodes.

We consider a Markov process $\textbf{Z}=(\textbf{Z}(t))_{t \ge 0}$, called the *aging process* on *G*, with

$$\textbf{Z}(t) = \left(Z_1(t ),\ldots ,Z_n(t)\right)$$

the network state at time *t*, where $Z_i(t) \in \{0,1\}$ is the state of node *i* at time *t*. The evolution of the nodes is as follows. At time *t*, node *i* goes from the healthy state to the damaged state, and vice versa, at rates, respectively,

2 $$\begin{aligned} \Gamma _+ (i,t) = A_+(f_i(t)), \qquad \Gamma _- (i,t) = A_-(f_i(t)), \end{aligned}$$

where

3 $$\begin{aligned} f_i (t) = \frac{1}{|N(i)|} \sum _{j \in N (i)} 1_{Z_j(t) = 1}, \end{aligned}$$

is the fraction of damaged neighbours of node *i* at time *t*, and

4 $$\begin{aligned} A_+(f) = \Gamma _0\,\textrm{e}^{r_+ f}, \qquad A_-(f) = \frac{\Gamma _0}{R} \,\textrm{e}^{-r_- f}, \qquad f \in [0,1], \end{aligned}$$

are two functions that play a key role throughout the sequel. Here, $r_+,r_-,\Gamma _0,R \in (0,\infty )$ play the role of *tuning parameters*. The exponential forms in (2) will be motivated in Sect. "Underlying network and poissonisation". Think of $\Gamma _0$ as the evolution rate for the network as a whole, and of *R* as tuning an asymmetry between the healthy state and the damaged state. Note that $\textbf{Z}$ has *time-dependent* transition rates, i.e., it is a *time-inhomogeneous Markov process*.

We label the *mortality nodes* by 1, 2, the *aging nodes* by $3,\dots ,n$, and define

$$\begin{aligned} S_\mathrm{{predeath}}&= \big \{z \in \{0,1\}^n:\,(z_1,z_2) \in \{(1,0),(0,1)\}\big \},\\ S_\mathrm{{death}}&= \big \{z \in \{0,1 \}^n:\,(z_1,z_2)=(1,1)\big \},\\ S_\mathrm{{other}}&= \{0,1 \}^n \setminus ( S_\mathrm{{predeath}} \cup S_\mathrm{{death}} ) = \big \{z \in \{0,1\}^n:\,(z_1,z_2) = (0,0) \big \} . \end{aligned}$$

Initially, all nodes are healthy, i.e.,

$$\textbf{Z}(0) = (0,\ldots ,0),$$

and the aging process $\textbf{Z}$ can move into and out of *every* state. The life time of the individual terminates when $\textbf{Z}$ enters $S_\mathrm{{death}}$, i.e., at time

5 $$\begin{aligned} \tau = \inf \left\{t \ge 0:\,\textbf{Z}(t) \in S_\mathrm{{death}}\right\}. \end{aligned}$$

The choice to use *two* mortality nodes merits further discussion. This choice was adopted in^16^, which introduced the above network model. In^28^ the possibility of a different (small) number of mortality nodes was explored numerically, and it was found that two nodes are optimal. In^13^ we carry out a systematic analysis of the optimal number of nodes for signal networks, and find that for most networks two nodes are optimal.

### Mortality rate

We are interested in the *mortality rate* at time *t* given by

6 $$\begin{aligned} m(t) = \lim _{\Delta \downarrow 0} \frac{1}{\Delta } \mathbb {P}(\tau \le t+\Delta \mid \tau \ge t) = - \frac{1}{s(t)} \frac{\textrm{d}}{\textrm{d}t} s(t), \end{aligned}$$

where

7 $$\begin{aligned} s(t) = \mathbb {P}(\tau >t) \end{aligned}$$

is the probability to survive up to time *t*. It is easily checked that Gompertz law implies

$$s(t) = C\, \textrm{e}^{-\frac{\alpha }{\beta } \textrm{e}^{\beta t}},$$

with *C* an integration constant. In particular, the density of the lifetime distribution equals

$$\frac{\textrm{d}}{\textrm{d}t}(1-s(t)) = C\, \alpha \, \textrm{e}^{\beta t - \frac{\alpha }{\beta } \textrm{e}^{ \beta t}}.$$

Given that $\textbf{Z}(t) = z \in S_\mathrm{{predeath}}$ with $(z_1,z_2)=(1,0)$, the probability that within time $0 < \Delta \ll 1$ the transition to $S_\mathrm{{death}}$ is made before any of the aging nodes changes equals $F(z)\Delta + o(\Delta )$ with

$$F(z) = \frac{A_+(\hat{f}_2(z))}{\sum _{i=1}^n 1_{z_i=0}\, A_+(\hat{f}_i(z)) + \sum _{i=1}^n 1_{z_i=1}\, A_-(\hat{f}_i(z))},$$

where

$$\begin{aligned} \hat{f}_i (z) = \frac{1}{|N(i)|} \sum _{j \in N (i)} 1_{z_j = 1}. \end{aligned}$$

A similar formula holds when $(z_1,z_2) = (0,1)$ with in the numerator $A_+(\hat{f}_1(z))$. The mortality rate at time *t* therefore equals

$$m(t) = \mathbb {E}\big [F(\textbf{Z}(t))\,1_{\textbf{Z}(t) \in S_\mathrm{{predeath}}} \mid \tau > t \big ] .$$

Since this quantity depends on the structure of the health network and the parameters $r_+,r_-,\Gamma _0,R$, it is a challenging task to find out how it depends on *t*.

### Summary

The above model has two key ingredients:

- *Choice of network structure:*
  - Aging graph *G*, scale-free and disassortative.
  - Number of nodes *n*, degrees of the nodes *N*(*i*), $i = 1,\ldots ,n$.
- *Choice of network dynamics:*
  - Aging dynamics $\textbf{Z} = (\textbf{Z}(t))_{t \ge 0}$, time-inhomogeneous Markovian.
  - Overall rate $\Gamma _0$ and adjustment factor *R*.
  - Damage and recovery rates $r_+$, $r_-$.

## Mathematical analysis

### Underlying network and poissonisation

**Figure 3:**
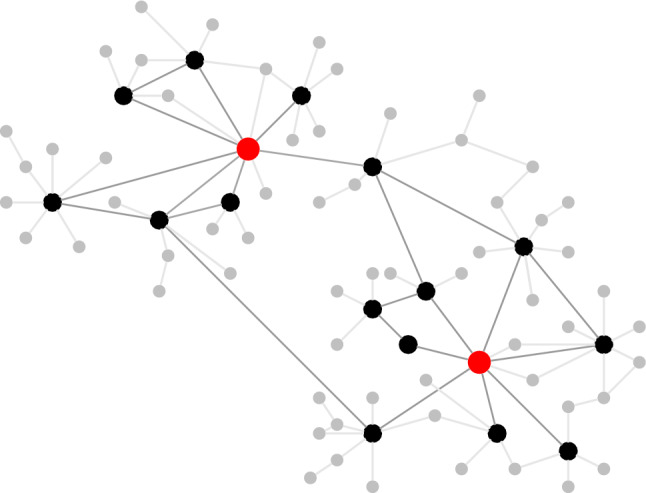
An example of an extended health network (compare with Fig. 2).

Below *G* lies a more complex and unobservable network, controlling the health of the individual. We view *G* as the observable resultant of this network at the level of health indicators, and $\textbf{Z}$ as the resultant of the dynamics through this network. In Fig. 3 the additional nodes of the more complex and unobservable network are depicted in grey (representing the ‘microscopic level’). These additional nodes act in clusters on each single black node (representing the ‘mesoscopic level’). While the nodes represent health indicators (such as blood pressure or cholesterol), the subnodes capture slowly changing background factors that influence these indicators.

We next argue why this choice of model is *not ad hoc*, but rather provides a *causal* approach to mortality, capturing both robustness and universality. In particular, we provide an explanation for why deficits and repairs accumulate at *exponential rates* at the node level as in (2). We show that this situation arises naturally when we interpret the state of an aging node or a mortality node (the observable black and red nodes) as representing the accumulated state of a large number of subnodes (the unobservable grey nodes). In other words, each node sees a *superposition* of processes at the subnodes.

The following theorem, taken from^2^, Theorem (10), says that *any* superposition of a large number of *sparse* point processes on $\mathbb {R}$ is close to a Poisson point process on $\mathbb {R}$. It does not need *any* assumptions on the distribution of the constituent point process other than that these are *thin* and *plenty*.

#### Theorem 1

**(Poisson)** For $n \in \mathbb {N}$, let $(M_k)_{k=1}^n$ be a sequence of independent point processes on $\mathbb {R}$ such that, for some measure $\mu$ on $\mathbb {R}$ and all finite intervals $I \subset \mathbb {R}$,

1. $$\lim _{n\rightarrow \infty } \sum _{k=1}^n \mathbb {P}(M_k(I) = 1) = \mu (I)$$,
2. $$\lim _{n\rightarrow \infty } \sup _{1 \le k \le n} \mathbb {P}(M_k(I) \ge 1) = 0$$,
3. $$\lim _{n\rightarrow \infty } \sum _{k=1}^n \mathbb {P}(M_k(I) \ge 2) = 0$$,

where $M_k(I)$ denotes the number of points that $M_k$ puts inside *I*. Then $M^n = \cup _{k=1}^n M_k$ converges weakly as $n\rightarrow \infty$ to the Poisson point process on $\mathbb {R}$ with intensity measure $\mu$.

Condition (1) guarantees that the superposition $M^n$ has an intensity measure $\mu _n$ that converges to a limiting intensity measure $\mu$. The interest is in the situation where $\mu$ is *non-trivial*, i.e., is neither zero nor infinity. Condition (2) says that the superposition is *infinitesimal*, i.e., each constituent point process makes a vanishing contribution. Condition (3) says that the superposition is *uniformly sparse*, i.e., points do not cluster. There is no (!) condition on the nature of the constituent point processes, which may have strong dependencies and may be far from being Poisson themselves. It is only through the *superposition* that the Poisson nature comes out in the limit, subject to the three conditions. For the special case where, for each $1 \le k \le n$, $M_k$ is a *stationary renewal process* with *mean interarrival time* $1/\lambda _k \in (0,\infty )$, condition (1) reads $\lim _{n\rightarrow \infty } \sum _{k=1}^n \lambda _k = \lambda \in (0,\infty )$, and the limit is the Poisson point process with intensity $\lambda$, i.e., exponentially distributed interarrival times with mean $1/\lambda$.

We use Theorem 1 with the role of space $\mathbb {R}$ taken over by time $[0,\infty )$. In that context the theorem says that the superposition of a large number of possibly time-dependent and mutually dependent clocks that ring rarely behaves like a single Poisson clock that rings at a possibly time-dependent rate.

### Mean-field and homogeneity assumptions

Now that we have set up the model, one way to proceed would be to make simple choices for *G*, like a *complete graph* with all the nodes connected, or a *bipartite graph* with the mortality nodes and the aging nodes forming two communities in the network, or a *scale-free graph* with the mortality nodes forming the hubs in the network and the aging nodes not being hubs. This is the approached followed by^16^ and the related literature discussed in the Introduction. Here we follow a new, analytical approach, which requires only very generic structural assumptions on the network. Results are derived by approximate computations based on a *mean-field assumption* and a *homogeneity assumption*. These assumptions are plausible because of the presumed hub structure of the mortality nodes. Below, whenever we write $\approx$ we refer to an approximation implied either by the two assumptions or by some other simplification. It remains a *mathematical challenge* to provide error bounds.

We approximate the dependence of a node on the fraction of damaged neighbours of that node, as expressed in (3), by the average of the nodes that are in state 1. To that end, we put

8 $$\begin{aligned} \hat{p}(t) = \frac{1}{n} \sum _{j=1}^n 1_{Z_j(t) = 1}, \qquad p(t) = \mathbb {E}[\hat{p}(t)] \end{aligned}$$

and we refer to *p*(*t*) as *damage fraction* at time *t*. For *n* large, the $Z_j(t)$ for nodes that are far apart are nearly independent, so that the average over the nodes acts like a *law of large numbers*.

- We make the following *mean-field assumption* (compare with (2)–(4)):

#### Assumption A1

The rates at time *t* for node *i* to go from healthy to damaged, respectively, from damaged to healthy are given by

$$\Gamma _+ (i,t) \approx A_+(p(t)), \qquad \Gamma _- (i,t) \approx A_-(p(t)),$$

with *p*(*t*) the average fraction of damaged nodes at time *t*. Note that the approximate rates do not depend on *i*. $\spadesuit$

- We comment on Assumption (A1). If the number of nodes *n* is large, and the neighbourhood *N*(*i*) of node *i* is large as well, then we can approximate (recall (3))
  9 $$\begin{aligned} f_i(t) = \frac{1}{|N(i)|} \sum _{j \in N (i)} 1_{Z_j(t) = 1} \approx \hat{p}(t) \approx p(t), \qquad i = 1,2. \end{aligned}$$

In a large *scale-free* and *disassortative* network there are a non-negligible number of nodes with high degrees, owing to the fat-tailed nature of the degree distribution. Indeed, there are predominantly many low-degree nodes but also a fair number of high-degree ones, which are connected to a significant fraction of the other nodes. As a result, the neighbourhoods of many nodes are large yet cover only a small fraction of the nodes of the entire network. The former ensures that the law of large numbers is in force, while the latter guarantees independence of the nodes. Combining (2) with (9), we approximate

10 $$\begin{aligned} \begin{aligned} \Gamma _+(i,t)&= A_+(f_i(t)) \approx \mathbb {E}[A_+(\hat{p}(t))] = \mathbb {E}\left[ \Gamma _0\,\textrm{e}^{r_+ \hat{p}(t)}\right] \approx \Gamma _0\,\textrm{e}^{r_+\mathbb {E}[\hat{p}(t)]} = A_+(p(t)),\\ \Gamma _-(i,t)&= A_-(f_i(t)) \approx \mathbb {E}[A_-(\hat{p}(t))] = \mathbb {E}\left[ \frac{\Gamma _0}{R}\,\textrm{e}^{-r_- \hat{p}(t)}\right] \approx \frac{\Gamma _0}{R}\,\textrm{e}^{-r_-\mathbb {E}[\hat{p}(t)]} = A_-(p(t)), \end{aligned} \end{aligned}$$

where in the second approximation we bring the expectation to the exponent (later we return to this simplification).

#### Remark 2

The health network introduced in^16^ also includes so-called *frailty nodes*: a small number of aging nodes that are highly connected. These frailty nodes allow for the modelling of the so-called *frailty index*^23^, which is of interest in health sciences. However, due to the mean-field approximation put forward in (9), according to which all nodes are treated in the same way, our approach does not allow for a closer analysis of the frailty nodes and the frailty index.

#### Remark 3

For short times, when few nodes are damaged, the law of large numbers that underlies the first approximation in (10) is less sharp than for large times, when many nodes are damaged. In the second approximation in (10) we brought the expectation to the exponent. However, by Jensen’s inequality for expectations of convex functions, we actually have

$$\mathbb {E}\left[ \textrm{e}^{r_+ \hat{p}(t)}\right] \ge \textrm{e}^{r_+\mathbb {E}[\hat{p}(t)]}, \qquad \mathbb {E}\left[ \textrm{e}^{-r_- \hat{p}(t)}\right] \ge \textrm{e}^{-r_-\mathbb {E}[\hat{p}(t)]},$$

i.e., the second approximation *undershoots* the two rates, in particular, the rate for a node to become damaged.

- We make the following *homogeneity assumption*:

#### Assumption A2

**Figure 4:**
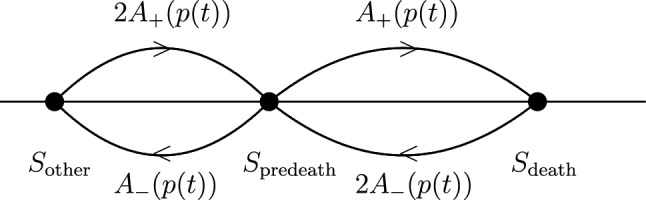
The aggregated time-inhomogeneous Markov chain.

The states in $S_\mathrm{{other}}$, $S_\mathrm{{predeath}}$, $S_\mathrm{{death}}$ are aggregated into three single states. The transition rates between these single states at time *t* are as in Fig. 4. $\spadesuit$

- We comment on Assumption (A2). The transition from $S_\mathrm{{other}}$ to $S_\mathrm{{predeath}}$ occurs when both two mortality nodes are healthy and one of them switches to damaged. The transition from $S_\mathrm{{predeath}}$ to $S_\mathrm{{death}}$ occurs when one mortality node is damaged, the other mortality node is healthy and switches to damaged. For the reverse transitions a similar argument applies. We note that direct transitions between $S_{\text {other}}$ and $S_{\text {death}}$ are excluded. Such a transition would require the simultaneous failure of both mortality nodes, which has zero probability due to the Poissonian nature of the underlying process.
- Combining the mean-field assumption and the homogeneity assumption, we obtain the following.

#### Lemma 4

Under Assumptions (A1)–(A2), *p*(*t*) is the solution of the autonomous differential equation

11 $$\begin{aligned} \frac{\textrm{d}}{\textrm{d}t}\, p(t) = (1-p(t))\,A_+(p(t)) - p(t)\,A_-(p(t)), \qquad p(0) = 0. \end{aligned}$$

#### Proof

The rate at which one of the $n(1-p(t))$ healthy nodes becomes damaged equals *n* times the first term, while the rate at which one of the *np*(*t*) damaged nodes becomes healthy equals *n* times the second term. The difference is the net rate at which the number of damaged nodes increases or decreases. Divide by *n* to get the fraction of damaged nodes. Note that $p(0)=0$ because initially all nodes are healthy. $\square$

### The approximate mortality rate

Our task is to derive the Gompertz law based on Assumption (A1) and Assumption (A2). In this section we derive a formula for the mortality rate. The argument proceeds in steps, listed as Lemmas 5–7 below, leading up to Theorem 8. In Sect. ”Analysis of the approximate mortality rate” we analyse this formula for several choices of the model parameters to get a feel for how the Gompertz law may emerge. We also derive a formula for the damage fraction at death, stated in Theorem 9 below.

#### Lemma 5

For every $$t \ge 0$$,

12 $$\begin{aligned} \begin{aligned} m(t)&= \lim _{\Delta \downarrow 0} \frac{1}{\Delta }\,\mathbb {P}(\tau \le t + \Delta \mid \tau \ge t)\\&= \lim _{\Delta \downarrow 0} \frac{1}{\Delta }\, \mathbb {P}\big (\exists \,0 \le u \le \Delta :\, \textbf{Z}(t+u) \in S_\mathrm{{death}} \mid \textbf{Z}(t) \in S_\mathrm{{predeath}}\big )\\&\qquad \times \mathbb {P}\big (\textbf{Z}(t) \in S_\mathrm{{predeath}} \mid \textbf{Z}(u) \not \in S_\mathrm{{death}}, 0 \le u \le t \big ). \end{aligned} \end{aligned}$$

#### Proof

Abbreviate

$$\begin{aligned} A_t&= \{\textbf{Z}(u) \not \in S_\mathrm{{death}}, 0 \le u \le t\},\\ B_t&= \{\textbf{Z}(t) \in S_\mathrm{{predeath}}\},\\ C_{t,\Delta }&= \{\exists \,0 \le u \le \Delta :\, \textbf{Z}(t+u) \in S_\mathrm{{death}}\}. \end{aligned}$$

Over short time intervals, $\textbf{Z}$ can only reach $S_\mathrm{{death}}$ by going from $S_\mathrm{{predeath}}$ to $S_\mathrm{{death}}$ in a single jump. Hence, for $\Delta \downarrow 0$,

$$\{t \le \tau \le t + \Delta \} \sim A_t \cap B_t \cap C_{t,\Delta },$$

where $\sim$ means equality up to an error that vanishes with $\Delta \downarrow 0$. Since

$$\{\tau \ge t\} = A_t,$$

it follows that, for $\Delta \downarrow 0$,

$$\begin{aligned} \mathbb {P}(\tau \le t+\Delta \mid \tau \ge t)&\sim \frac{\mathbb {P}(A_t \cap B_t \cap C_{t,\Delta } )}{\mathbb {P}(A_t)} = \frac{\mathbb {P}(A_t \cap C_{t,\Delta } \mid B_t)\,\mathbb {P}(B_t)}{\mathbb {P}(A_t)}\\&= \frac{\mathbb {P}(C_{t,\Delta } \mid B_t)\,\mathbb {P}(A_t \mid B_t)\,\mathbb {P}(B_t)}{\mathbb {P}(A_t)} = \mathbb {P}(C_{t,\Delta } \mid B_t)\,\mathbb {P}(B_t \mid A_t), \end{aligned}$$

where the second equality uses the Markov property at time *t*. $\square$

From the mean-field assumption we obtain the following representation of the first factor in (12).

#### Lemma 6

Under Assumption (A1),

$$\lim _{\Delta \downarrow 0} \frac{1}{\Delta } \mathbb {P}\big (\exists \,0 \le u \le \Delta :\, \textbf{Z}( u+t) \in S_\mathrm{{death}} \mid \textbf{Z}(t) \in S_\mathrm{{predeath}}\big ) \approx A_+(p(t)).$$

#### Proof

The rate to jump from a predeath state to the death state is approximately $A_+(p(t))$, because one mortality node is damaged, the other mortality node is healthy, and the latter needs to switch to damaged. $\square$

From the homogeneity assumption we obtain the following representation of the second factor in (12).

#### Lemma 7

Under Assumption (A2),

$$\mathbb {P}\big (\textbf{Z}(t) \in S_\mathrm{{predeath}} \mid \textbf{Z}(u) \not \in S_\mathrm{{death}}, 0 \le u \le t \big ) \approx \chi (t)$$

with $\chi (t)$ the solution of the differential equation

13 $$\begin{aligned} \frac{\textrm{d}}{\textrm{d}t}\,\chi (t) = (1-\chi (t))\,2A_+(p(t)) - \chi (t)\,A_-(p(t)), \qquad \chi (0) = 0. \end{aligned}$$

#### Proof

The law of the time-homogeneous Markov process $\textbf{Z}$ on the set of three states $\{S_\mathrm{{other}}, S_\mathrm{{predeath}}, S_\mathrm{{death}}\}$ *conditional* on not entering $S_\mathrm{{death}}$ is the same as the law of the time-homogeneous Markov process $\textbf{Z}'$ on the set of two states $\{S_\mathrm{{other}},S_\mathrm{{predeath}}\}$ where the rates to and from $S_\mathrm{{death}}$ are *set to zero* (see Fig. 4). Hence $\chi (t) = \mathbb {P}\big (\textbf{Z}'(t) \in S_\mathrm{{predeath}})$. This probability solves (13), because the net rate at which $\chi (t)$ increases is the difference of the rates at which $\textbf{Z}'$ jumps into, respectively, jumps out of the state $S_\mathrm{{predeath}}$. Note that $\chi (0)=0$ because initially all nodes are healthy. $\square$

Combining Lemmas 4–7, we can identify the approximate mortality rate.

#### Theorem 8

**(Approximate mortality rate)** Under Assumptions (A1)–(A2),

14 $$\begin{aligned} m(t) \approx A_+(p(t))\,\chi (t). \end{aligned}$$

The solution of (11) tells us how *p*(*t*) grows as a function of *t*. Once this profile has been determined, the solution of 13 tells us how $\chi (t)$ grows as a function of *t*. The two together fix how *m*(*t*) grows as a function of *t*.

A further interesting quantity is the *damage fraction at death*.

#### Theorem 9

**(Damage fraction at death)**

15 $$\begin{aligned} \mathbb {E}[\hat{p}(\tau )] \approx \int _0^\infty \textrm{d}t\, A_+(p(t))\,p(t)\,\chi (t)\,\exp \left[ -\int _0^t \textrm{d}u\, A_+(p(u))\,\chi (u)\right] . \end{aligned}$$

#### Proof

By (5), (7) and (8),

$$\mathbb {E}[\hat{p}(\tau )] \approx \int _0^\infty p(t)\,\mathbb {P}(\tau \in \textrm{d}t) = \int _0^\infty p(t) \left[ -\frac{\textrm{d}}{\textrm{d}t} s(t)\right] \,\textrm{d}t.$$

It follows from (6) that

$$s(t) = \exp \left[ -\int _0^t m(u)\,\textrm{d}u\right]$$

and hence

$$-\frac{\textrm{d}}{\textrm{d}t} s(t) = m(t)\,\exp \left[ -\int _0^t m(u)\,\textrm{d}u\right] .$$

Use (14) to get the claim. $\square$

### Analysis of the approximate mortality rate

Having derived an approximate formula for the mortality rate *m*(*t*) in terms of the differential equations in (11) and (13), our next task is to see whether this formula produces the Gompertz law for times that are neither too small nor too large.

We first *scale time* by $1/\Gamma _0$ so as to remove the parameter $\Gamma _0$. Putting

16 $$\begin{aligned} p^*(s) = p\left( \frac{s}{\Gamma _0}\right) , \qquad \chi ^*(s) = \chi \left( \frac{s}{\Gamma _0}\right) , \qquad m^*(s) = m\left( \frac{s}{\Gamma _0}\right) , \end{aligned}$$

and

17 $$\begin{aligned} A^*_+(p) = \textrm{e}^{r_+ p}, \qquad A^*_-(p) = \frac{1}{R}\,\textrm{e}^{-r_- p}, \qquad p \in [0,1], \end{aligned}$$

we can rewrite (14) as

18 $$\begin{aligned} m^*(s) \approx \Gamma _0\,A^*_+(p^*(s))\,\chi ^*(s), \end{aligned}$$

where $p^*(s)$ and $\chi ^*(s)$ solve the differential equations (recall (11) and (13))

19 $$\begin{aligned} \begin{array}{lll} & \frac{\textrm{d}}{\textrm{d}s}\, p^*(s) = (1-p^*(s))\,A^*_+(p^*(s)) - p^*(s)\,A^*_-(p^*(s)), & p^*(0) = 0,\\[0.3cm] & \frac{\textrm{d}}{\textrm{d}s}\, \chi ^*(s) = (1-\chi ^*(s))\,2A^*_+(p^*(s)) - \chi ^*(s)\,A^*_-(p^*(s)), & \chi ^*(0) = 0. \end{array} \end{aligned}$$

Note that $\Gamma _0$ drops out of (19) because

20 $$\begin{aligned} t = \frac{s}{\Gamma _0} \quad \longrightarrow \quad \frac{\textrm{d}}{\textrm{d}s} = \Gamma _0 \frac{\textrm{d}}{\textrm{d}t}, \end{aligned}$$

so that a factor $\Gamma _0$ can be cancelled on both sides of the differential equations. Think of *t* as the ‘microscopic’ time scale on which the single nodes in the health network evolve, and of *s* as the ‘macroscopic’ time scale on which the network consisting of many nodes evolves as a whole. If $\Gamma _0 = \frac{1}{C}/\textrm{year}$, then one unit of macroscopic time corresponds to *C* years of human aging (think of *C* as a ‘calibration parameter’.)

Since $p^*(s)$ and $\chi ^*(s)$ are differentiable in *s*, they are continuous in *s* as well. Note that $p^*(s)$ is strictly increasing in *s* with $\lim _{s\rightarrow \infty } p^*(s) = p_\dagger$, where $p_\dagger$ solves the equation $(1-p_\dagger )\,A^*_+(p_\dagger ) = p_\dagger \,A^*_-(p_\dagger )$, so

21 $$\begin{aligned} p_\dagger = \frac{A^*_+(p_\dagger )}{A^*_+(p_\dagger )+A^*_-(p_\dagger )} = \frac{1}{1 + \frac{1}{R}\, \textrm{e}^{-(r_+ + r_-)p_\dagger }}. \end{aligned}$$

Note that also $\chi ^*(s)$ is strictly increasing in *s* with $\lim _{s\rightarrow \infty } \chi ^*(s) = \chi _\dagger$, where $\chi _\dagger$ solves the equation $(1-\chi _\dagger )\,2A^*_+(p_\dagger ) = \chi _\dagger \, A^*_-(p_\dagger )$, so

22 $$\begin{aligned} \chi _\dagger = \frac{2A^*_+(p_\dagger )}{2A^*_+(p_\dagger )+A^*_-(p_\dagger )} = \frac{2p_\dagger }{1+p_\dagger }. \end{aligned}$$

It is further evident that $\frac{\textrm{d}}{\textrm{d}s} p^*(0) = 1$ and $\frac{\textrm{d}}{\textrm{d}s} \chi ^*(0) = 2$. For later use we need the following observation.

#### Lemma 10

$$p^*(s)< \chi ^*(s) < 2p^*(s)$$ for all $$s > 0$$.

#### Proof

Put

$$\begin{aligned} \Delta _1(s) = \chi ^*(s) - p^*(s), \qquad \Delta _2(s) = 2p^*(s) - \chi ^*(s). \end{aligned}$$

It follows from (19) that

$$\begin{aligned} \begin{aligned} \frac{\textrm{d}}{\textrm{d}s} \Delta _1(s)&= - \big [A^*_+(p^*(s)) + A^*_-(p^*(s))\big ]\, \Delta _1(s) + A^*_+(p^*(s))\,(1-\chi ^*(s)),\\ \frac{\textrm{d}}{\textrm{d}s} \Delta _2(s)&= - A^*_-(p^*(s))\, \Delta _2(s) + 2A^*_+(p^*(s))\,\Delta _1(s). \end{aligned} \end{aligned}$$

We know that $\Delta _1(0)=0$, $\Delta _1(s)>0$ for small $s>0$, and $\Delta _1(s)$ is continuous and differentiable in *s*. It is impossible that $\Delta _1(s)$ hits the value 0 at some $s_1>0$, because this would imply that $\frac{\textrm{d}}{\textrm{d}s} \Delta _1(s_1) = A^*_+(p^*(s_1))\,(1-\chi ^*(s_1)) > 0$. Hence $\Delta _1(s)$ is everywhere strictly positive. Similarly, we know that $\Delta _2(0)=0$, $\Delta _2(s)>0$ for small $s>0$, and $\Delta _2(s)$ is continuous and differentiable in *s*. It is impossible that $\Delta _2(s)$ hits the value 0 at some $s_2>0$, because this would imply that $\frac{\textrm{d}}{\textrm{d}s} \Delta _2(s_2) = 2A^*_+(p^*(s_2))\,\Delta _1(s_2) > 0$. Hence $\Delta _2(s)$ is everywhere strictly positive as well. $\square$

**Figure 5:**
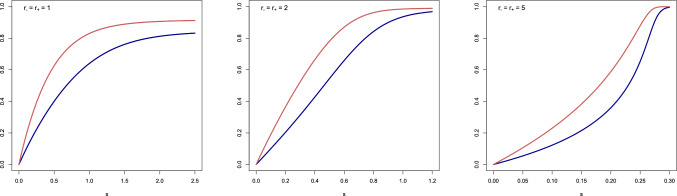
Plots of $p^*(s)$ (= blue curve) and $\chi ^*(s)$ (= red curve) for $r_+ = r_- \in \{1,2,5\}$ and $R=1$.

**Figure 6:**
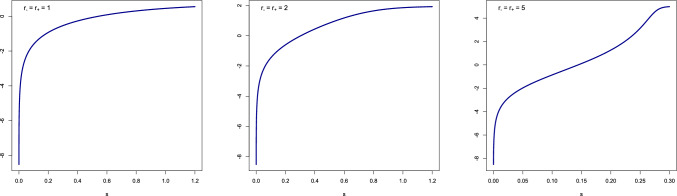
Plots of $\ln [A^*_+(p^*(s))\,\chi ^*(s)]$ for $r_+ = r_- \in \{1,2,5\}$ and $R=1$.

Figures 5–6 show plots of $p^*(s)$, $\chi ^*(s)$ and $\ln [A^*_+(p^*(s))\,\chi ^*(s)]$ for $r_+ = r_- \in \{1,2,5\}$ and $R=1$ (carried out with the help of the programming language R). The latter curve is linear only for values of *s* that are neither too small nor too large. The *range* of *s*-values for which the linear fit is accurate depends on the *choice* of the parameters. The curves for $p^*(s)$, $\chi ^*(s)$ tend to be concave for small values of $r_+,r_-$ and convex-concave for large values of $r_+,r_-$. The curve for $\ln [A^*_+(p^*(s))\,\chi ^*(s)]$ tends to be concave for small values of $r_+,r_-$ and concave-convex-concave for large values of $r_+,r_-$.

## Simulations and numerical results

While the aging process $\textbf{Z}$ is well-defined for any choice of parameters $n,r_+,r_-,\Gamma _0,R$ with $n\in \mathbb {N}$ and $r_+,r_-,\Gamma _0,R \in (0,\infty )$, it only provides a fair model for the mortality rate when the parameters are chosen properly. In this section we discuss an instance of the aging process provided in^16^, where the parameters were chosen to be $n = 10^4$, $r_+ = 10.27$, $r_- = 6.5$, $R = 1.5$ and $\Gamma _0 = 0.00113$/year. For this choice of $\Gamma _0$, one unit of *s* corresponds to 885 years, and so [40, 100] years corresponds to $s \in [0.045,0.113]$.

**Figure 7:**
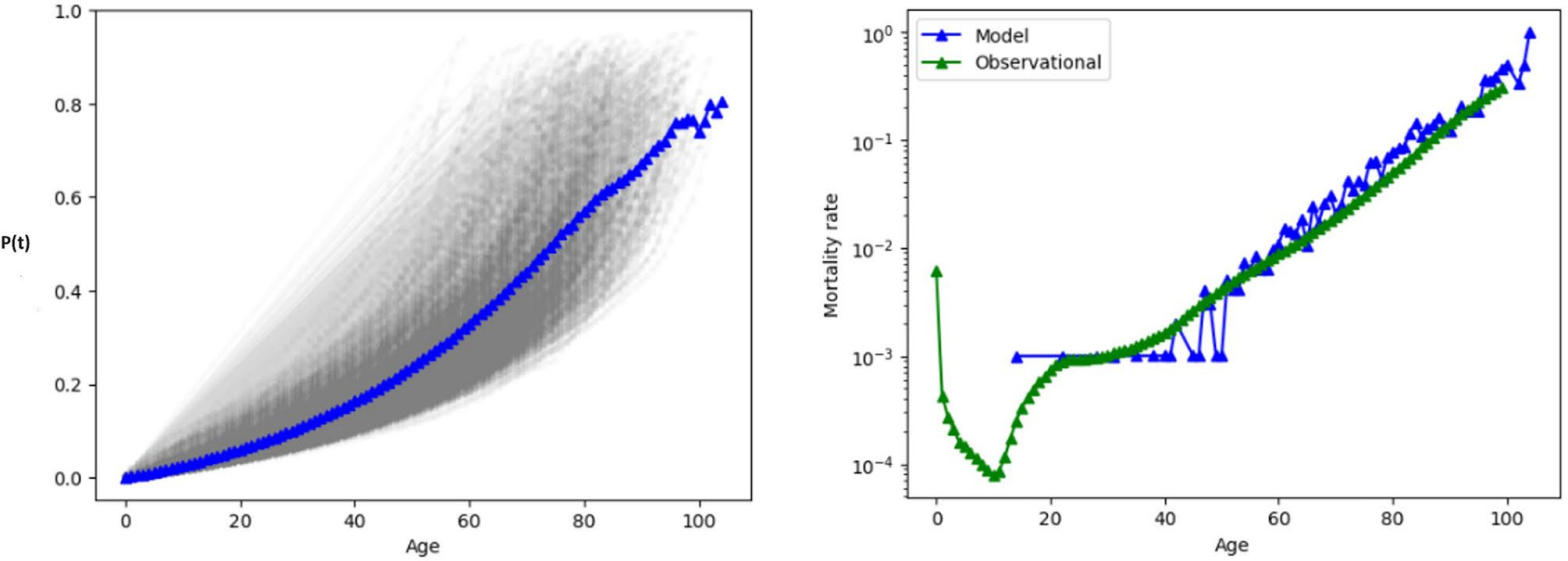
A simulation of the damage fraction and the mortality rate for the parameter choices in^16^. *Left:* The black curves are realisations of the damage fraction, the blue curve is the average damage fraction. *Right:* The blue curve is the result of simulation of the network dynamics, the green curve represents the statistical data obtained from Fig. 1.

Figure 7 plots the damage fraction and the mortality rate in the range between 0 to 100 years of age. For the latter, ‘observational’ means taken from the statistical data for US men in the period 2010-2019^17^ (recall Fig. 1), while ‘model’ means taken from simulation of the *network dynamics* based on $10^6$ i.i.d. samples^8^, Chapter 6. It is seen that $\ln m(t)$ is roughly linear over the age interval [40, 100] years, which confirms Gompertz law *numerically*. It is also shown that the exponential increase of mortality is consistent with a linear increase of the fraction of damaged nodes *p*(*t*), i.e. a linear accumulation of damage at the level of health indicators.

The fitted line is $\ln m(t) \approx -9.76 + 0.085\,t$, which gives

$$\alpha \approx \textrm{e}^{-9.76} \approx 5.8 \times 10^{-5}, \qquad \beta \approx 8.5 \times 10^{-2}.$$

**Figure 8:**
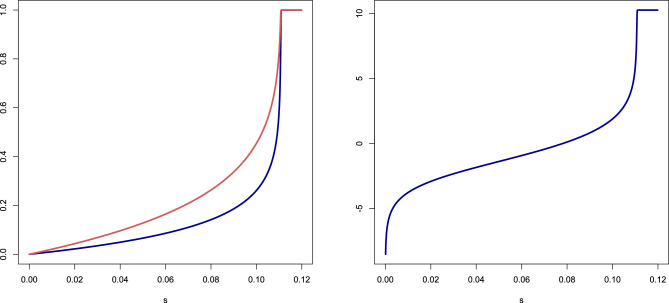
Plots for $r_+ = 10.27$, $r_- = 6.5$, $R = 1.5$. *Left:* Plots of $p^*(s)$ (= blue curve) and $\chi ^*(s)$ (= red curve). *Right:* Plot of $\ln [A^*_+(p^*(s))\chi ^*(s)]$.

Figure 8 shows plots of $p^*(s)$, $\chi ^*(s)$ and $\ln [A^*_+(p^*(s))\chi ^*(s)]$ based on (18)–(19) for the values of $r_+,r_-,R$ used in^16^. The latter curve is roughly linear on the interval $s \in [0.02, 0.08]$, but bends down on the left of this interval and bends up on the right.

**Figure 9:**
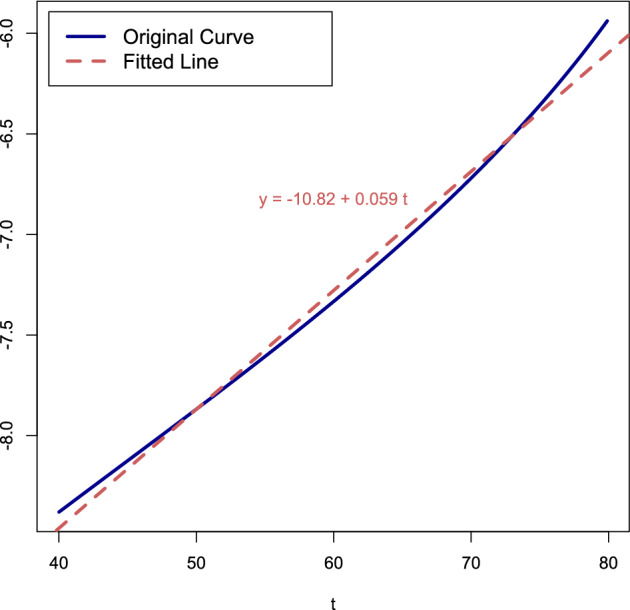
Plot of $\ln m(t)$ for $t \in [40,80]$ years, for $r_+ = 10.27$, $r_- = 6.5$, $R = 1.5$, $\Gamma _0 = 0.00113$/year (= blue curve). The best fitted line (= dashed red line) corresponds to the Gompertz law.

Figure 9 shows a plot of $\ln m(t)$ for $t \in [40,80]$ years based on (16) and (18)–(19) for the values of $r_+,r_-,R,\Gamma _0$ used in^16^. The best fitted line as the linear regression fit is $\ln m(t) \approx -10.82 + 0.059\,t$, which gives

$$\alpha \approx \textrm{e}^{-10.82} \approx 2.0 \times 10^{-5}, \qquad \beta \approx 5.9 \times 10^{-2}.$$

The value of $\alpha$ is about $34\%$ of the value in Fig. 1, the value of $\beta$ is about $69\%$ times the value in Fig. 1. Thus, the match is fairly good.

### Remark 11

The linear regression fit depends on the *t*-interval that is chosen. For instance, Table 1 shows the results obtained by fitting linear regression lines to the data points on the curve $\ln m(t)$ at different time ranges.

**Table 1:** Properties of best fitted lines.

*t*-interval	$\alpha$	$\beta$	R-squared	MSE
[40, 80] years	$\approx 2.0 \times 10^{-5}$	$\approx 5.9 \times 10^{-2}$	0.99	0.00
[40, 85] years	$\approx 1.6 \times 10^{-5}$	$\approx 6.3 \times 10^{-2}$	0.99	0.01
[40, 90] years	$\approx 1.2 \times 10^{-5}$	$\approx 6.8 \times 10^{-2}$	0.98	0.02
[40, 95] years	$\approx 7.1 \times 10^{-6}$	$\approx 7.8 \times 10^{-2}$	0.94	0.10
[40, 100] years	$\approx 8.3 \times 10^{-7}$	$\approx 1.1 \times 10^{-1}$	0.67	1.89

As observed in Table 1, choosing a longer time interval decreases the value of $\alpha$ and increases the value of $\beta$. However, the decrease in *R*-squared and the increase in the mean-squared error MSE indicate that the fitted line is diverging from the curve. The reason is that the curve in Fig. 9 bends up. Thus, Theorem 8 only yields a somewhat *crude form* of the Gompertz law.

### Remark 12

A numerical estimation of $\mathbb {E}[\hat{p}(\tau )]$ in (15) based on Monte-Carlo integration produces a value between 0.66 and 0.70 for the parameter values used in^16^. Hence, roughly and on average, a fraction $\frac{2}{3}$ of the nodes is damaged when death occurs.

## An analytic approximation

The first differential equation in (19) is non-linear and does *not* admit a closed form solution for every choice of the parameters. The second differential equation in (19) is linear given the solution of the first and can therefore be solved explicitly:

$$\chi ^*(s) = \int _0^s \textrm{d}u\,2A^*_+(p^*(u))\,\exp \left[ -\int _u^s \textrm{d}v\,\left[2A^*_+(p^*(v)) + A^*_-(p^*(v))\right]\right] .$$

Both differential equations are not hard to solve *numerically*, as shown in Sect. ”Simulations and numerical results”, but it is natural to ask whether it is possible to find an *analytic approximation* of $p^*(s)$, $\chi ^*(s)$ and $\ln \left[A^*_+(p^*(s))\,\chi ^*(s)\right]$. Below we give an affirmative answer. Any formula that expresses these quantities directly in terms of the parameters $r_+,r_-,R$, even in an approximate form, is helpful for a better understanding of the evolution of the damage fraction and the mortality rate.

The differential equation for $p^*(s)$ in (19) can be integrated to give

$$s = \int _0^{p^*(s)} \frac{\textrm{d}\lambda }{(1-\lambda )\,A^*_+(\lambda ) - \lambda \,A^*_-(\lambda )},$$

where we use that $p^*(0)=0$, noting that the integrand is integrable near $\lambda = 0$. In order to obtain a formula for $p^*(s)$ as a function of *s*, the expression needs to be inverted. We proceed by deriving upper and lower bounds.

Lower bound: Estimate

$$\begin{aligned} s&= \int _0^{p^*(s)} \frac{\textrm{d}\lambda \,\textrm{e}^{-r_+\lambda }}{(1-\lambda ) - \frac{\lambda }{R}\,\textrm{e}^{-(r_+ + r_-)\lambda }} \le \int _0^{p^*(s)} \frac{\textrm{d}\lambda \,\textrm{e}^{-r_+\lambda }}{(1-\lambda ) - \frac{\lambda }{R}}\\&\le \frac{1}{1-\frac{R+1}{R}\,p^*(s)} \int _0^{p^*(s)} \textrm{d}\lambda \,\textrm{e}^{-r_+\lambda } = \frac{1}{1-\frac{R+1}{R}\,p^*(s)}\,\frac{1}{r_+}\left[ 1-\textrm{e}^{-r_+ p^*(s)}\right] \le \frac{p^*(s)}{1-\frac{R+1}{R}\,p^*(s)}. \end{aligned}$$

Inverting this inequality, we get

$$p^*(s) \ge \frac{s}{1+\frac{R+1}{R}\,s}.$$

Since $\chi ^*(s) \ge p^*(s)$ by Lemma 10, it follows from (18) that

$$m^*(s) \gtrapprox \frac{\Gamma _0\,s}{1+\frac{R+1}{R}\,s} \exp \left[ \frac{r_+ s}{1+\frac{R+1}{R}\,s}\right] ,$$

which via (16) becomes

23 $$\begin{aligned} m(t) \gtrapprox \frac{\Gamma _0^2\,t}{1+\frac{R+1}{R}\,\Gamma _0\,t} \exp \left[ \frac{r_+ \Gamma _0\,t}{1+\frac{R+1}{R}\,\Gamma _0\,t}\right] , \end{aligned}$$

In case $\frac{R+1}{R}\,\Gamma _0\,t \ll 1$, the right-hand side simplifies to $\Gamma _0^2\,t\,\textrm{e}^{r_+\Gamma _0\,t}$.

Upper bound: Estimate

$$s \ge \int _0^{p^*(s)} \textrm{d}\lambda \,\textrm{e}^{-r_+\lambda } = \frac{1}{r_+}\left[ 1-\textrm{e}^{-r_+ p^*(s)}\right] .$$

Inverting this inequality for *s* that $r_+ s<1$, we get

$$p^*(s) \le - \frac{1}{r_+} \ln (1-r_+ s) = \frac{1}{r_+} \sum _{k=1}^\infty \frac{(r_+ s)^k}{k}.$$

Since $\chi ^*(s) \le 2 p^*(s)$ by Lemma 10, it follows from (18) that

$$m^*(s) \lessapprox \frac{2\Gamma _0}{r_+} \left[ \sum _{k=1}^\infty \frac{(r_+ s)^k}{k}\right] \exp \left[ \sum _{k=1}^\infty \frac{(r_+ s)^k}{k}\right] ,$$

which via (16) becomes

24 $$\begin{aligned} m(t) \lessapprox \frac{2\Gamma _0}{r_+} \left[ \sum _{k=1}^\infty \frac{(r_+ \Gamma _0\,t)^k}{k}\right] \exp \left[ \sum _{k=1}^\infty \frac{(r_+ \Gamma _0\,t)^k}{k}\right] . \end{aligned}$$

In case $r_+\Gamma _0\,t \ll 1$, the right-hand side simplifies to $2\Gamma _0^2\,t\,\textrm{e}^{r_+\Gamma _0\,t}$.

The bounds in (23)–(24) show that, as long as both $\frac{R+1}{R}\Gamma _0\,t \ll 1$ and $r_+ \Gamma _0\,t \ll 1$, a fair approximation for $\beta$ in the Gompertz law is

25 $$\begin{aligned} \beta \approx r_+\Gamma _0, \end{aligned}$$

while $\alpha$ can be crudely sandwiched for $t\in [40,80]$ as

26 $$\begin{aligned} 40\,\Gamma _0^2 \lessapprox \alpha \lessapprox 160\,\Gamma _0^2. \end{aligned}$$

The bounds in (26) are a factor 4 apart.

**Figure 10:**
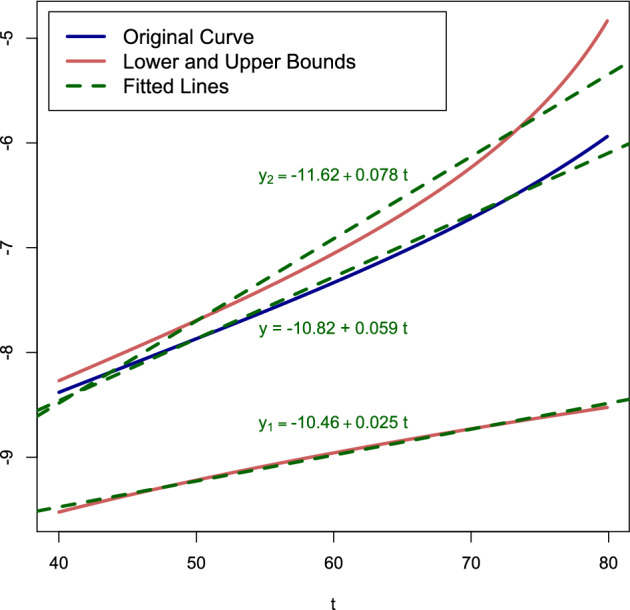
Plot of $\ln m(t)$ for $t \in [40,80]$ years for the parameter values used in^16^. The middle curve (= blue curve) is the one in Fig. 9. The bottom curve and the top curve (= red curves) represent the numerical plot of the logarithm of the lower bound in (23) and the upper bound in (24). The best fitted lines (= green dashed curves) correspond to the Gompertz law.

For the parameter values used in^16^, Fig. 10 compares the curve in Fig. 9 with the bounds obtained in (23)–(24). It seems that the upper bound in (24) is better than the lower bound in (23). The slopes of the best fitted lines are $7.8 \times 10^{-2}$ for the upper bound, $2.5 \times 10^{-2}$ for the lower bound, and $5.9 \times 10^{-2}$ for the true curve. Thus, the values of $\beta$ obtained from the bounds are off by about 50%, which is fair but not sharp. Interestingly, the values of $\ln \alpha$ in the three cases differ by not more than 8%, which is rather sharp.

For the parameter values used in^16^, $\frac{R+1}{R}\Gamma _0\,t \approx 0.075$ and $r_+ \Gamma _0 t \approx 0.46$ at $t=40$ years. The former is small, the latter is not, which makes the approximations in (25)–(26) a priori questionable. The approximation in (25) would predict $\beta \approx 1.2 \times 10^{-2}$, which is below the true value by a factor of about 5. The approximation in (26) would predict $-9.88 \lessapprox \ln \alpha \lessapprox -8.50$, which interval lies above the true value. The lower bound of the interval is off by about 9%, the upper bound by about 20%. Again, the approximation of $\alpha$ is better than that of $\beta$.

## Conclusion

The main contributions of the present paper are the following.

1. We have provided *mathematical* arguments to support the *network* description of aging and mortality proposed in^16^.
2. We have argued that Poisson rates for the evolution of the network are *universal* as long as the mechanism according to which nodes switch between healthy and damaged is “the net result of many small influences”.
3. With the help of two assumptions, valid for *scale-free* and *disassortative* networks, we have shown that the *Gompertz law* holds approximately in a certain age range. The precise formula for the mortality rate *deviates* somewhat from the Gompertz law, but we have shown that the deviation is small. The size of the network plays no role after the two assumptions have been implemented, which again underlines the *universality* of the network description.
4. We have linked the parameters in the Gompertz law to the parameters driving the evolution of the network. For the curves in Figs. 1 and 7 we have found a *fair fit* with the model parameters used in^16^.
5. We have found that the fraction of damaged nodes is the solution of a non-linear differential equation, which is easy to handle numerically but hard to solve analytically. While this solution is in general not linear in time, the numerical solution shows that it behaves *approximately linearly* over the age range 20 to 80 years, as shown in Fig. 8.
6. We have further found that the mortality rate involves the solution of two non-linear differential equations. Exact bounds on the solutions of these differential equations allow us to derive a *crude* analytic approximation of the mortality rate that is *explicit* in the model parameters.
7. The nodes in our health network (see Fig. 2) can be interpreted as representing organs in the body. However, a more appropriate interpretation is that they represent *health indicators*, such as blood pressure, blood sugar level, cholesterol level, etc. A node is in a healthy state when the corresponding indicator falls inside the clinically normal range, and is in a damaged state when it falls outside. The indicators are influenced by many factors in the background, which tend to change slowly (see Fig. 3). Of course, the mathematical theory does not depend on what the nodes represent.
8. The states of the single nodes switch over time, but the average fraction of damaged nodes grows with time. Gompertz law is consistent with a linear accumulation of damage of the health indicators found in molecular measurements.

Finally, it is well known that the Gompertz law is not perfect. Yet, it is ubiquitous, simple and widely regarded as a good approximation of the *empirical law* for the mortality rate as a function of age^19^. The Gompertz law is known to be relevant in many species, from yeast to fruit flies, from dogs to horses. For instance, mice accumulate health deficits just like humans^20^. Thus, the network description of aging via the accumulation of health deficits *in principle* covers a wide range of species, with parameters varying across species.

## Data Availability

The data used in this paper is available in the Human Mortality Database, https://www.mortality.org/.
